# Aurora kinases: novel therapy targets in cancers

**DOI:** 10.18632/oncotarget.14893

**Published:** 2017-01-29

**Authors:** Anqun Tang, Keyu Gao, Laili Chu, Rui Zhang, Jing Yang, Junnian Zheng

**Affiliations:** ^1^ Jiangsu Center for the Collaboration and Innovation of Cancer Biotherapy, Cancer Institute, Jiangsu, China; ^2^ Department of Oncology, The First Affiliated Hospital, Xuzhou Medical University, Xuzhou, Jiangsu, China

**Keywords:** Aurora kinases, mitosis, cancer therapy target, Aurora kinases inhibitors, combination therapy

## Abstract

Aurora kinases, a family of serine/threonine kinases, consisting of Aurora A (AURKA), Aurora B (AURKB) and Aurora C (AURKC), are essential kinases for cell division *via* regulating mitosis especially the process of chromosomal segregation. Besides regulating mitosis, Aurora kinases have been implicated in regulating meiosis. The deletion of Aurora kinases could lead to failure of cell division and impair the embryonic development. Overexpression or gene amplification of Aurora kinases has been clarified in a number of cancers. And a growing number of studies have demonstrated that inhibition of Aurora kinases could potentiate the effect of chemotherapies. For the past decades, a series of Aurora kinases inhibitors (AKIs) developed effectively repress the progression and growth of many cancers both *in vivo* and *in vitro*, suggesting that Aurora kinases could be a novel therapeutic target. In this review, we'll first briefly present the structure, localization and physiological functions of Aurora kinases in mitosis, then describe the oncogenic role of Aurora kinases in tumorigenesis, we shall finally discuss the outcomes of AKIs combination with conventional therapy.

## INTRODUCTION

Mitosis controlling the mother cells to divide into two daughter cells with equal chromosomes and cytoplasm is accurately regulated by a series of serine/threonine kinases in cell cycle, and among which Aurora kinases are important and indispensable in multiple steps of mitotic progression. The three members of Aurora kinases family [1] hold high homogeneity in mammalian cells. In term of the role of mitotic regulators, deletion of AURKA caused mitotic spindle assembly and chromosome segregation failure, subsequently resulted in genetic instability and a significantly increased tumor incidence [2–4]. In addition, Aurora kinases deficiency also caused polyploid oocytes in mice [5], and early embryonic lethality has been observed in *AURKA−/−* mice [2, 6]. Loss of AURKB activity could override spindle assembly checkpoint (SAC) through premature removal of SAC proteins from the kinetochore [7], which leads to defect of chromosome segregation and conformation of polyploidy. Overexpression or amplification of Aurora kinases is generally detected in amount of human cancers, such as breast cancer [8–11], ovarian cancer [12–14], gastric/gastrointestinal cancer [15, 16] and other tumors [11, 17–33] (Table 1) and is associated with the poor prognosis [8, 34, 35]. Thus, Aurora kinases become promising therapeutic targets and numerous AKIs have been developed. In present review, we outline the recent progresses along with the emerging obstacles associated with Aurora kinases in cancers.

**Table 1:** Summary of Aurora kinases and Aurora kinases inhibitors in clinical trails

Kinases	Localization	Function	Tumors types	Inhibitors and clinical trials
AURKA	Centrosome, Spindle microtubule, Midbody	Centrosome maturation/separation; Mitotic entry; Microtubule nucleation; Spindle assembly; Bipolar spindle microtubule formation; Cytokinesis; Mitosis exit	Breast cancer[8, 9]; Ovarian cancer[12–14]; Gastric/Gastrointestinal cancer[15, 16]; Colorectal cancer[17]; Esophageal squamous cell carcinoma[20, 21]; Lung cancer[22]; Cervical cancer[24]; Prostate cancer[11, 26]; Glioma[28]; Acute myeloid leukemia(AML)[30]; Oral cancer[32]	MLN8054; phase I[123] ENMD-2076; phase II[124] MLN8237; phase III[125, 126] AT9283; phase I/II[129, 130] VX-680/MK-0457; phase II[131] PHA-680632; preclinical[132] AMG-900; phase I[133] PHA-739358; phase II[134, 135] CYC-116; phase I[136]
AURKB	Chromosome Kinetochore Midbody	Chromosome condensation; Microtubule-kinetochore attachment; Chromosomal alignment; Chromosomal segregation; Regulating SAC Cytokinesis	Breast cancer[10]; Ovarian cancer[14]; Gastric/Gastrointestinal cancer[15]; Colorectal cancer[18]; Lung cancer[23]; Cervical cancer[24]; Prostate cancer[27]; Glioma[28, 29]; Acute myeloid leukemia(AML)[31]; Oral cancer[33]	AZD1152; phase II /III[127, 128] AT9283; phase I/II[129, 130] VX-680/MK-0457; phase II[131] PHA-680632; preclinical[132] AMG-900; phase[133] PHA-739358; phase II[134, 135] CYC-116; phase I[136]
AURKC	Chromosome Midbody	Meiotic chromosome segregation; Similar to AURKB, e.g. Cytokinesis	Breast cancer[11] ; Colorectal cancer[19]; Cervical cancer[25]; Prostate cancer[11]; Glioma[29];	VX-680/MK-0457; phase II[131] PHA-680632; preclinical[132] AMG-900; phase I[133] PHA-739358; phase II[134, 135] CYC-116; phase I[136]

## STRUCTURE, LOCALIZATION AND FUNCTIONS OF AURORA KINASES IN MITOSIS

### Structure of Aurora kinases

**Figure 1:**
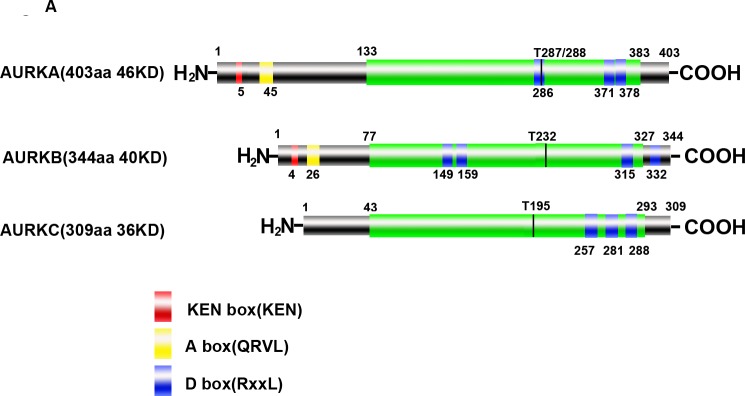

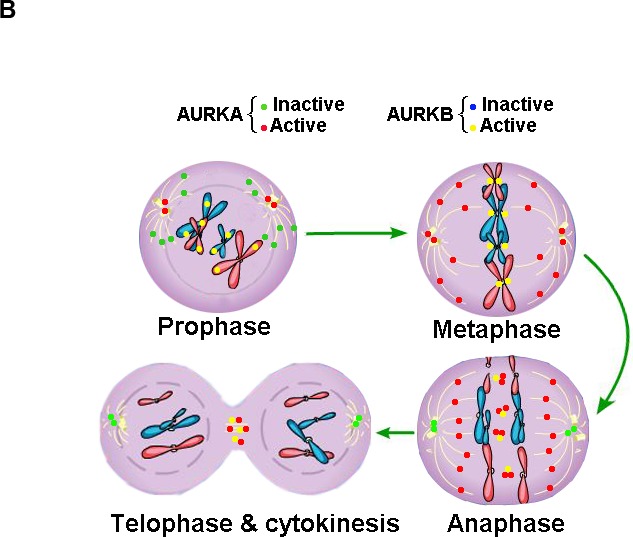
Structure and cellular distribution of Aurora kinases in mitosis **A**. Schematic drawing of AURKA, AURKB and AURKC proteins with indicated domains. **B**. Cellular localization shift of Aurora kinases in mitosis (AURKC is not shown due to the elusive cellular localization and function).

Aurora kinases are highly conserved and hold homologous structure, constituting of a N-terminal domain, a protein kinase domain and C-terminal domain [36–39]. High consistency among Aurora kinases is found in the catalytic domain of the C-terminal containing catalytic T-loop and degradation box (A-box/D-box/KEN-box). Under physical mitosis, Aurora kinases can be targeted and activated by several protein cofactors including target protein for *Xenopus* kinesin-like protein 2 (TPX2) and inner centromere protein (INCENP). Additionally, each kinase of Aurora family members is activated through auto-phosphorylation on catalytic T-loop residues which are Thr288 (AURKA), Thr232 (AURKB) and Thr195 (AURKC), respectively (Figure 1A). Upon dephosphorylation mediated by protein phosphatase 1(PP1), the activities of Aurora kinases become inactive [40]. In the late mitosis, Aurora kinases are recognized by anaphase-promoting complex/cyclosome (APC/C) and subsequently degraded.

### Localization of Aurora kinases

AURKA localizes to the duplicate centrosomes from the beginning of S phase and shifts to the bipolar spindle microtubules during mitosis, finally, moves to perinuclear materials of the daughter cell at the end of mitosis [41]. By contrast, AURKB starts at early G2 and localizes to the chromosomes in prophase, the centromere in prometaphase and metaphase, the central spindle in anaphase and the mid-body in cytokinesis [42]. Recent study identified that AURKC localized to centrosome in the interphase and binded to chromosome during mitosis [43]. However, the exact distribution shift of AURKC during the mitosis is still non-established (Figure 1B). Based on their distinct subcellular localizations during mitosis (Table 1), the functions of Aurora kinases are distinguished and summarized in Table 1.

### Functions of Aurora kinases

Once localizing to centrosome, AURKA is activated by LIM protein ajuba, and the expression and activity of AURKA arrives peak at G2/M transition, stimulating duplicated centrosomes to separate at G2/M transition and initiating the mitotic entry. Activated AURKA recruits several pericentriolar proteins including γ-tubulin and TACC/MAP215 [44, 45] to microtubule organizing center (MTOCs) which facilitates centrosome maturation and speedy microtubule nucleation in eukaryotic cell. After nuclear membrane breaks down in prometaphase, AURKA is activated, targeted to microtubule by TPX2 [46, 47], and required for spindle assembly and the conformation of bipolar spindle microtubule [48]. At the end of the mitosis, AURKA is degraded by cadherin-1(Cdh1)/APC/C complex [49], and mitotic exist.

AURKB is a component of chromosome passenger complex (CPC), composing of additional three activation regulators INCEP, survivin and borealin [50–53]. It mediates chromosome condensation by phosphorylating histone H3 on Ser10 and variant centrosome protein A (CENP-A) on Ser7 [54]. AURKB is also involved in regulating SAC, rectifying the faulty attachment between spindle and kinetochore, maintaining the correct chromosome alignment and the faithful chromosomal segregation. Most recent study demonstrated that activated AURKB mediated phosphorylation of Histone H2AX at Ser121, which in turn promoted the auto-phosphorylation of AURKB, forming a positive feedback and further accelerating AURKB activation [55]. During anaphase, AURKB phosphorylates a series of downstream substrates, including mitotic kinesin-like protein 1 (MKLP1) and RacGAP1 [56], facilitates their deposition at mid-body, and maintains the stabilization of central spindle. Moreover, AURKB could phosphorylate Kif2A, the microtubule de-polymerase, leading to shorten of central spindle, and promote cytokinesis [57].

Unlike AURKA and AURKB, AURKC is specifically expressed in mammalian testis compared to other somatic tissues [58]. Forced-expression of mutant AURKC in mouse oocytes causes oocytes cell cycle arrest at meiosis I and formulating eggs of aneuploidy, implicating that AURKC exerts pivotal role in meiotic chromosome segregation [59]. Since AURKC is required as part of the CPC [60, 61], AURKC has overlapping functions with AURKB in mitosis [62, 63]. Recent study demonstrated that AURKC interacted with transforming acidic coiled-coil 1 (TACC 1) and co-localized to the mid-body of Hela cells during cytokinesis [64].

## THE ROLES OF AURORA KINASES IN CANCER

AURKA, B and C are mapped on intrinsic unstable with frequent defection, amplification and mutations regions of 20q13.2, 17p13.1 and 10q13, respectively [65–67], giving a good explanation of abnormal expression of Aurora kinases in human cancers. So far, no reports have shown that natural deficiency of Aurora kinases in human tumors. However, amount of studies have demonstrated that the overexpression or amplification of Aurora kinases has been observed in various human cancers as showed in Table 1, and several types of Aurora kinases mutations have been detected in different somatic cancer samples, including lung, colorectal and melanoma [62], which indicates that Aurora kinases play a determinant role in cell transformation and oncogenesis. For the past decades, increasing researches focus on these potential oncogenic proteins about how they function in tumor development.

### AURKA controls proliferation, epithelial-mesenchymal transition (EMT) and metastasis, as well as self-renewal capacity of cancer stem cells (CSCs)

Recently, it's demonstrated that AURKA is overexpressed, distributed beyond the nucleus in cancer cells [68] and involved in the tumorigenesis through multiple mechanisms. AURKA could phosphorylate RAS-association domain family 1, isoform A (RASSF1A), a novel tumor suppressor, disrupt RASSF1A-mediated microtubules stabilization and M-phase cell cycle arrest, and lead to uncontrolled proliferation in cancers [69]. AURKA also phosphorylates IκBα, an inhibitor of NF-κB, hence activating NF-κB signaling pathway. Besides, AURKA could contribute to cancer cell survival *via* increasing anti-apoptotic modulators (Bcl-2 [70], MCL-1 [71]) and decreasing pro-apoptotic regulators (Bax [70], Bim [72], PUMA [73]) or suppressing autophagy through activating mammalian Target of Rapamycin (mTOR) signaling [74]. Moreover, overexpression of AURKA inhibits the release of cytochrome c (cyt c) from mitochondria, suppressing the formation of apoptotic body with Apaf-1 and apoptosis [75].

Patients with poor prognosis are normally due to metastasis mediated by EMT. AURKA upregulates the expression of SLUG [76], an EMT transcription factor, and fibrillin 1 (FBN1) that is an important fibrillin regulating microenvironment [77]. Meanwhile, AURKA induces a decrease in the expression of E-cadherin and β-catenin which play a pivotal role in regulating cell-cell adhesion, and consequently promotes EMT [78]. AURKA also activates Wnt and Akt signaling pathway simultaneously *via* reducing H3K4/H3K27 methylation on the promoter of Twist, a famous negative factor of MET, and then promotes EMT [79]. Also, other constitutive activation of oncogenic signalings, such as Raf-1 [80], Myc [81], OCT4 [82], promote the EMT progression *via* the stabilization and accumulation of AURKA. In addition, AURKA overexpression can significantly enhance the expression of matrix metalloproteinases (MMP)-2, MMP-7 and MMP-10, leading to degradation of extracellular matrix proteins, which stimulates tumor cell mobility and metastasis [20, 83].

Interestingly, overexpression of AURKA has been detected in CSCs [84] harboring the characteristics of self-renewal and differentiation into all cell types. Recent study confirmed that AURKA could activate Wnt signaling pathways in Glioma-initiating cells (GIC) *via* interacting with AXIN and stabilizing β-catenin, thereby promoting the ability of GICs to self-renewal [85]. On the other hand, the β-catenin/TCF4 complex could in turn transcriptionally activate AURKA and subsequently inhibit Glycogen synthase kinase 3β (GSK3β), which further stabilizes β-catenin. Therefore, AURKA/Wnt signal pathway forms a positive regulation loop and strengthens the expression of core CSCs.

### AURKB promotes cell cycle and survival of cancer cells

Overexpression or amplification of AURKB has been clarified in various human tumors, which implies that it contributes to tumorigenesis in spite of its non-established role of oncogene.

Beside the aneuploidy resulting from AURKB overexpression, it's also implicated in promoting cell cycle *via* inhibiting or enhancing cell cycle-related targets. AURKB decreases the expression of the cell cycle inhibitor p21^WAF1/CIP1^ *via* suppressing p53 activity [86], resulting in aberrant activation of Cyclin-dependent kinase 1 (Cdk1), leading to cell cycle progression and thereby promoting cell survival. Additionally, Cdk1 activates the acetyltransferase TIP60, causing acetylation and activation of AURKB, which further contributes to abnormal aneuploidy and uncontrolled cell cycle progression. In contrast to TIP60, histone deacetylases (HDACs) govern the opposite function that decrease the ability of histones to bind to DNA and thereby globally mediate transcriptional repression. However, Guise AJ et al identified that AURKB induced cell cycle progression through regulating its substrate Class IIa HDACs [87]. In consistence with Guise AJ group, it has been shown that AURKB and HDACs synergistically regulate cell survival and proliferation in lymphoma *via* activating AKT/mTOR signaling pathway [88].

Moreover, inhibition of AURKB by a selected inhibitor Barasertib (AZD1152-HQPA) induces apoptosis and necrosis in metastatic melanoma [89]. Inhibition of AURKB decreases the expression of Cyclin B1 and Cyclin D1, and elevates the Caspase 3 expression in lymphoma cells [88]. All of these data indicate that aberrant expression of AURKB might induce the cell survival-associated proteins expression and decrease the expression of pro-apoptosis proteins, although the precise molecular mechanism remains elusive.

### AURKC may promote tumor progression

Given the highly expression in gametes, AURKC is required to regulate chromosome segregation during meiosis. Chromosome mis-segregation during meiosis due to aberrant AURKC expression or activity leads to aneuploidy in gametes, causing genetic infertility [90]. AURKC may promote tumor development in view of overlapping and complementary function with AURKB, as well as gene amplification and overexpression in cancers [11] though the mechanism is still in dispute.

## AURORA KINASES FORM COMPLEX NETWORK WITH THEIR REGULATORS IN TUMORIGENESIS

Beside the well-established overexpression of Aurora kinases, Aurora kinases can collaborate with numerous protein including tumor suppressors and oncogenes and promote carcinogenesis.

### Aurora kinases downregulate p53 and form feedback loop with p53

It's well-established that AURKA can regulate p53, the well-known tumor suppressor, through phosphorylation on both Ser215 and Ser315 residues [91, 92], which inhibits p53 transcriptional activity and enhances Mdm2-mediated p53 degradation, respectively. Interestingly, AURKA also inhibits p53 activity *via* phosphorylating heterogeneous nuclear ribonucleoprotein K (hnRNPK) on Ser379, a transcriptional coactivator of p53 required for p53 activation in the occurrence of gene damage [93]. Similarly, AURKB can suppress p53 transcriptional activity through forming complex with novel inhibitor of histone acetyltransferase repressor (NIR) and p53, in which NIR functions as a scaffold protein to mediate AURKB localization to the DNA binding domain (DBD) of p53 and then phosphorylates p53 on Ser269 and Thr284 in DBD. Recently, another study proved that AURKB also directly interacted with p53 *via* phosphorylating p53 on Ser183, Thr211 and Ser215, similar to AURKA [94]. So far, no interaction has been reported between p53 and AURKC.

**Figure 2:**
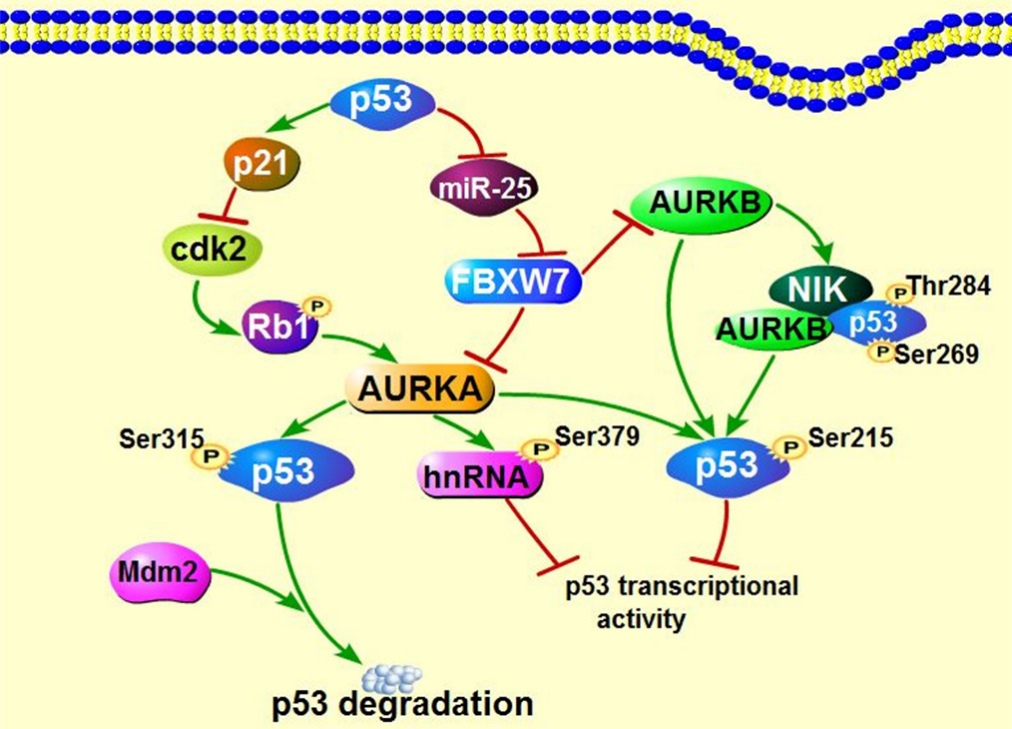
Interaction of Aurora kinases and tumor suppressor p53 Both AURKA and AURKB can regulate p53 through directly or indirectly mechanisms as indicated above. Moreover, the p53 deficiency which is often shown in numerous cancers further contributes to the expression of Aurora kinases, facilitating tumor development.

In a feedback loop, p53 inhibits AURKA *via* both transcriptional and posttranscriptional regulation [95]. Silencing of p53 increases Cdk2 activity by reducing p21^wAF1/CIP1^ expression, in turn Cdk2 phosphorylates Rb1 and dissociates E2F3, which then promotes AURKA expression *via* binding to its gene promoter [95]. In addition, Mutant p53 or loss of p53 functions causes elevated expression of miR-25 expression and leads to a decreased in level of F-box and WD repeat-containing 7 (FBXW7), a E3 ubiquitin ligase and well-known tumor suppressor, resulting in AURKA overexpression [96]. In agreement with AURKA, it's demonstrated that FBXW7 is also a negative regulator for AURKB, the mutant FBXW7 leads to upregulation of AURKB as well [97]. Taken together, any deviation in FBXW7/AURKB/p53 feedback loop could contribute to tumorigenesis and accelerate tumor progression. Intriguingly, AURKC overexpression gives rise to polyploidy and excessive centrosome, which can be aggravated in the absence of p53 [62], indicating p53 deficiency facilitates the role of AURKC in tumorigenesis. Since the Aurora kinases aberrant expression and p53 mutation are sometimes simultaneously detected in cancers, it is hard to clarify which one happens originally. It's possible that Aurora kinases coordinates with p53 to control tumorigenesis (Figure 2).

### Aurora kinases suppress the functions of breast cancer susceptibility proteins (BRCA)

**Figure 3:**
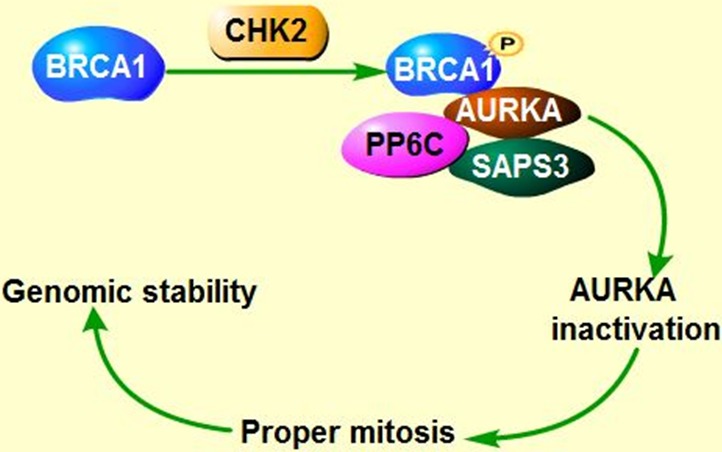

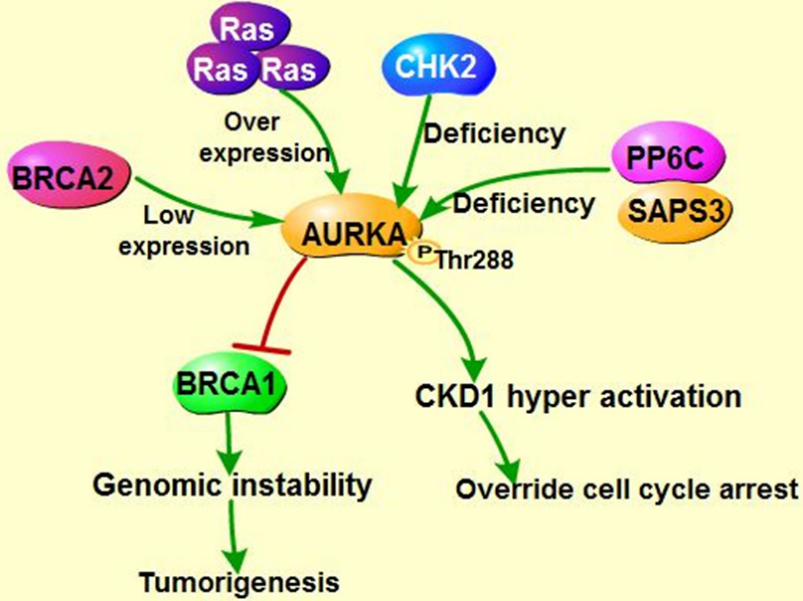
Co-regulation of AURKA, BRCA and Ras in tumorigenesis **A**. CHK2-BRCA1 axis suppress AURKA oncogenic function. **B**. AURKA inactivates Tumor suppressor BRCA1, leading to genomic instability and contributing to cell transformation. In addition, AURKA overexpression along with BRCA2 mutation facilitates the cell cycle *via* hyper-phosphorylating cell cycle kinase CDK. Imbalance between AURKA and BRCA2 could enhance Ras-mediated tumorigenesis.

As known, checkpoint kinase 2 (CHK2)-BRCA1 tumor suppressor axis restrained AURKA oncogenic function [98] through recruiting serine/threonine protein phosphatase 6 catalytic subunit and regulatory subunit site 4-associated protein 3 (PP6C-SAPS3) phosphatase to AURKA catalytic T-loop, ensuring proper mitosis. However, unleashed AURKA activity resulting from the loss of CHK2 or PP6C-SAPS3 could in turn phosphorylate and inactivate BRCA1, which exacerbates chromosomal instability and contributes to tumorigenesis [99]. Tumor suppressor BRCA2 is also involved in maintaining genomic stability and suppressing polyploidy [100] (Figure 3A). Previous study demonstrated that AURKA is commonly overexpressed in breast cancers with *BRCA2* mutation [101] and overexpressed AURKA could also suppress BRCA2 in ovarian cancer, indicating that there exists a negative correlation between AURKA and BRCA2 [13]. In circumstance of BRCA2 mutation, overexpressed AURKA might hyper-activate Cdk1 through phosphorylation of cell division cycle phosphatase 25B (CDC25B) on Ser353, leading to override of cell cycle arrest, contributing to cells transformation and tumorigenesis [101, 102] (Figure 3B). Therefore, AURKA has been regarded as a prognostic marker of breast cancer arising from BRCA2 mutation [101, 103]. However, the precise regulation mechanisms between AURKA and BRCA2 require further study. Intriguingly, both AURKA and BRCA2 are the downstream targets of RAS, overexpressed RAS abates BRCA2 expression but induces overexpression of AURKA, which in turn could increase the expression of farnesyl protein transferase β (FTβ), enhancing oncogene *RAS-*induced tumorigenesis *via* promoting RAS farnesylation [104] (Figure 3B).

### Aurora kinases can be up-regulated by Myc and other signaling pathways

**Figure 4:**
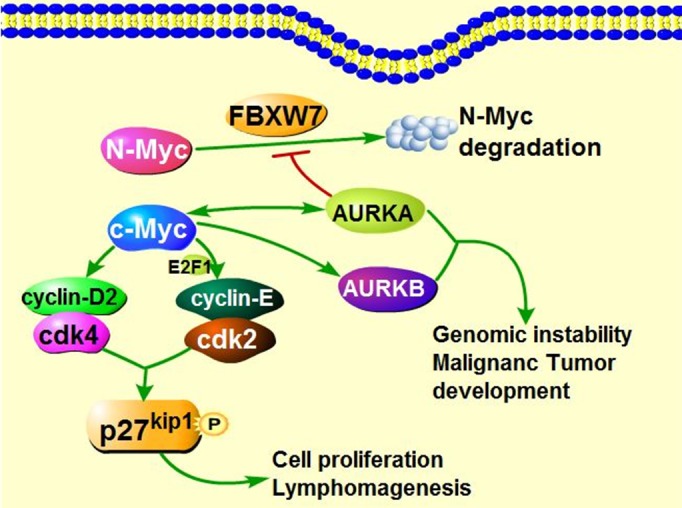
The interaction of Myc and Aurora Kinases in tumorigenesis AURKA can transcriptionally upregulates the expression of c-Myc. On the other hand, c-Myc also binds to promoter of AURKA and transcriptionally increases the expression of AURKA. Additionally, activated c-Myc up-regulates the expression of cyclin D2, cdk4 as well as cyclin-E, contributing to the formation of cyclin-E/cdk2 complex, phosphorylating p27^Kip1^ on Thr187, and consequently could provoke the cell proliferation and induce the Myc-mediated lymphomagenesis. c-Myc indirectly transcriptionally activate AURKB through unclear mechanisms. Besides, AURKA protects N-Myc from FBXW7-mediated degradation. High expression of AURKA and AURKB are implicated in tumorigenesis.

AURKA also mediates Myc (N-Myc, c-Myc, L-Myc) oncogenic effects in cancers. Overexpression or activation of Myc and AURKA are commonly simultaneously detected in human cancers. AURKA can function as a Myc regulator *via* binding to the CCCTCCCCA motif in the NHE III1 region and promote *c-Myc* transcription [105]. Conversely, c-Myc can transcriptionally up-regulate AURKA through binding to *AURKA* promoter [106], forming a positive regulation loop. Moreover, the *c-Myc* activation further leads to cell cycle-related genes transcription, which enhances cell proliferation and Myc-induced lymphomagenesis [107, 108]. In addition, AURKA interacts with N-Myc and protects N-Myc from FBXW7-mediated degradation [109, 110]. Opposite to AURKA, c-Myc could indirectly promote AURKB transcription though the unclear mechanisms [106] (Figure 4).

Besides the oncogenes or tumor suppressors, Aurora kinases are also regulated by other oncogenic proteins such as protein kinase C (PKC), thereby contributing to tumorigenesis. Aurora kinases are the cofactor in PKC-mediated oncogenesis in which the activation of PKC induces the mitogen-activated protein kinase (MAPK)-mediated phosphorylation of AURKA and AURKB, leading to activation of NF-κB/AP-1, consequently promoting the migration and invasion [111]. Moreover, our previous study confirmed that oncoprotein BCR-ABL can elevate expression of AURKA and AURKB through Akt signaling pathway in chronic myelogenous leukemia (CML) [112]. Studies demonstrated AURKA as a direct target of *β*-Catenin [113, 114]. Interestingly, AURKA can enhance *β*-Catenin activity *via* inactivating *β*-Catenin negative regulator GSK3*β* [115, 116], leading to increased level of *β*-Catenin nuclear translocation and transcriptionally activity [117, 118].

## OUTCOME OF AKIS IN CLINICAL TRIALS

**Figure 5:**
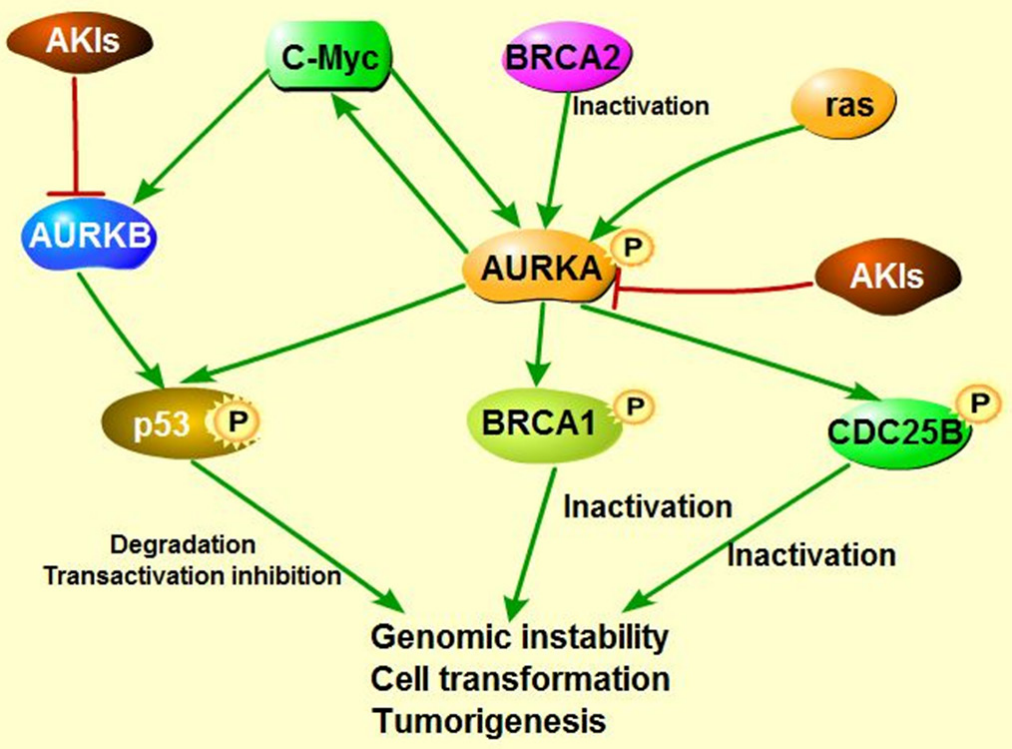
The model of AKIs targeting into several signaling pathways Inhibition of Aurora kinases by AKIs could not only suppress the pro-oncogenic function, but also could block tumorigenesis *via* several signaling pathways.

Given overexpression or gene amplification of Aurora kinases has been identified in diverse cancers, making them become potent targets of cancer therapy, a series of AKIs have been produced for the past decades and inhibition of expression or activity of Aurora kinases by AKIs indeed suppresses cell proliferation, migration and invasion in cancer cells [29, 119, 120], inhibits the progress and growth of many cancers [95, 121, 122] as Figure 5 shown, and more exciting is that some AKIs have already been used into clinical trials [123–136] (Table 1). Based on current researches and observations, MLN8237 (Alisertib), one of AURKA selective inhibitor, and the AURKB selective inhibitor AZD1152 are successfully attracted researchers attention and are undergoing III clinical trials due to their potential dominant suppression for cancer treatment [125, 127].

In this part, we will take MLN8237 as an example to address the clinical outcome. A phase II study of MLN8237 [125] with multiple advanced solid tumors has been finished. The dosage of MLN8237 was orally at 50 mg, twice daily for 7 days followed by a break of 14 days in 21-day cycles. Partial responses were observed in nine patients (18%) of 49 women with breast cancer, ten patients (21%) of 48 participants with small-cell lung cancer (SCLC), one (4%) of 23 patients with non-small-cell lung cancer (NSCLC), four (9%) of 45 patients with squamous cell cancer of head and neck (SCCHN), and four (9%) of 47 people with gastro-esophageal adenocarcinoma. In another phase II trial, patients with platinum-resistant or refractory epithelial ovarian, fallopian tube carcinomas, or primary peritoneal carcinoma were treated with MLN8237. The median progression-free survival (PFS) was 1.9 months. Interestingly, sixteen out of 31 patients achieved stable disease with a mean duration of response of 2.86 months. These observations suggest that MLN8237 has modest single-agent antitumor activity and may produce responses and durable disease control [137].

## COMBINATION WITH AKIS OVERCOMES CONVENTIONAL RESISTANCE IN CANCERS

A subset of AKIs have been developed, unfortunately, up to now, no AKIs have been proved for clinical use on patients and some obstacles we should take into consideration such as cell toxicity [138, 139]. Thus, AKIs that specifically and efficiently target cancer cells but not the normal cells should be developed in the future to reduce toxicity. A new study [140] demonstrated that AZD2811, an AURKB inhibitor, can be formulated in a nanoparticle named accurin to attenuate the side effect and increase the efficacy of AZD2811 in mouse tumor xenograft models, which may give a new direction for the development of AKIs.

Interestingly, increasing researches have uncovered that Aurora kinases are prone to confer cancer cell radio- and chemo-resistance. Recent research identified that AURKA was overexpressed in NSCLC and contributed to cisplatin-based chemotherapy resistance [141]. Consistently, AURKB overexpression also induces the tamoxifen resistance and poor prognosis in breast cancer [10, 142]. Additionally, it has been shown that AURKB conferred cancer cell resistance to tumor necrosis factor-related apoptosis-inducing ligand (TRAIL)-induced apoptosis *via* phosphorylating survivin [143]. Taken together, Aurora kinases could become novel predictors in cancers prognosis, and simultaneously inhibition of Aurora kinases could overcome the drug resistance or/and enhance the anti-tumor effect of traditional compounds. Therefore, we herein mainly discuss the most recent progress of combination of AKIs and other cancer target therapy in cancers.

Tyrosine kinase inhibitors (TKIs) substantially ameliorated the outcome of CML, however, patients with BCR-ABL mutations have a poor response to TKIs, especially, *T315I BCR-ABL* mutation is even resistant to the second generation TKIs nilotinib and dasatinib, which actually can inhibit most BCR-ABL mutations [144, 145]. Notably, recent study addressed that inhibition of AURKA sensitized mutant BCR-ABL even T315I mutant CML cells to both generation TKIs, [146]. Additionally, another study showed that dual inhibition of BCR-ABL and AURKB could also suppressed proliferation and induced apoptosis of mutant BCR-ABL cells [147].

Opyrchal M et al firstly demonstrated that AURKA activated SMAD5 oncogenic signaling pathway and thereby down-regulated estrogen receptor α (ERα), leading to estrogen resistance in ERα^+^ breast cancers and combination tamoxifen with MLN8237 abrogated the endocrine resistance [148]. Moreover, Inhibition of AURKB with AZD1152 inhibited the proliferation of estrogen-resistant breast cancer cells [149], which also sheds light on the overcome the estrogen-resistance breast cancer.

It has been shown that AURKA was involved in platinum-resistance and administration of either VX-680 or MLN8237 re-sensitized cancer cells to platinum and attenuated the migration ability of platinum-resistant NSCLC cells [141]. Besides, a group recently demonstrated that daurinol, a novel topoisomerase inhibitor, can inhibit the transcriptional activity of both AURKA and AURKB, which increased the radio-sensitivity of tumor cells *in vivo* and *in vitro* [150].

Sequential AURKB inhibition with AZD1152 synergistically enhanced the inhibitory effect of cisplatin in cisplatin-resistant ovarian cancer *via* downregulating c-Myc [151]. Furthermore, AURKA inhibitor PHA680632 [152] and MLN8237 [153], AURKB inhibitor AZD1152 [154] enhanced tumor response to radiotherapy in p53-deficient cancer cells, atypical teratoid/rhaboid tumors and androgen-resistant prostate cancer respectively. Overexpression of brain derived neurotrophic factor (BDNF) was associated with cisplatin-resistant neuroblastoma (NB) [155], combination chemotherapy with Aurora kinases inhibitor, PHA-680632, suppressed the transcription of BDNF 5′UTR exons 1, 2c, 4 and potentiated the cytotoxic effect of cisplatin in NB cell [156]. In addition, a combination of pan-AKIs R763 and EGFR antibody cetuximab activated cell cycle checkpoint and induced apoptosis in cetucimab-resistant SCCHN [157].

Given the involvement in conventional cancer therapy resistance, Aurora kinases are considered as predictors of chemotherapy response and prognosis [158, 159]. AKIs in combination with conventional modality, including chemotherapy and radiotherapy, could effectively inhibit tumor development and provide a promising new therapeutic strategy for individuals with cancer.

## CONCLUSIONS AND PROSPECTIVE

Aurora kinases become promising therapeutic targets in cancers, however, the serious challenge is failure to distinguish the normal cells which acquire the physiological function of Aurora kinases and thereby gives rise to high toxicity, indicating that targeting Aurora kinases is likely a double-edged sword. Moreover, multiple researches have addressed that the participation of Aurora kinases in chemo-resistance in conventional therapy. However, inhibition of Aurora kinases somehow rescues chemo-resistance, which sheds light on cancer therapy through combination chemotherapy. We need to pay more concentration to further unearth Aurora kinases’ complicated roles in tumorigenesis, and further studies discovering potential targets correlated to Aurora kinases and involved in tumorigenesis may broaden our eyes to invent new compounds and therapeutic strategy.

## Abbreviations

AKIs: Aurora kinases inhibitors
SAC: spindle assembly checkpoint
APC/C: anaphase-promoting complex/cyclosome
TPX2: *Xenopus* kinesin-like protein 2
INCEP: inner centromere protein
PP1: protein phosphatase 1
MTOCs: microtubule organizing center
Cdh1: cadherin-1
CPC: chromosome passenger complex
CENP-A: centrosome protein A
MKLP1: mitotic kinesin-like protein 1
TACC 1: transforming acidic coiled-coil 1
EMT: epithelial-mesenchymal transition
CSCs: cancer stem cells
RASSF1A: RAS-association domain family 1, isoform A
mTOR: mammalian Target of Rapamycin
cyt c: cytochrome c
FBN1: fibrillin 1
MMP: matrix metalloproteinases
GIC: Glioma-initiating cells
GSK3β: Glycogen synthase kinase 3 beta
Cdk: Cyclin-dependent kinase
HDACs: Histone Deacetylases
hnRNPK: heterogeneous nuclear ribonucleoprotein K
NIR: Novel inhibitor of histone acetyltransferase repressor
DBD: DNA binding domain
FBXW7: F-box and WD repeat-containing 7
BRCA: breast cancer susceptibility gene
CHK2: checkpoint kinase 2
PP6C-SAPS3: serine/threonine protein phosphatase 6 catalytic subunit and regulatory subunit site4-associated protein 3
CDC25B: cell division cycle phosphatase 25B
FTβ: farnesyl protein transferase β
PKC: protein kinase C
MAPK: mitogen-activated protein kinase
PKB: protein kinase B
CML: chronic myelogenous leukemia
SCLC: small-cell lung cancer
NSCLC: non-small-cell lung carcinoma
SCCHN: squamous cell cancer of head and neck
PFS: progression-free survival
TRAIL: tumor necrosis factor-related apoptosis-inducing ligand
AML: acute myeloid leukemia
TKIs: tyrosine kinase inhibitor
ERα: estrogen receptor alpha
BDNF: brain derived neurotrophic factor
NB: neuroblastoma

## References

[1] Nigg EA (2001). Mitotic kinases as regulators of cell division and its checkpoints. Nat Rev Mol Cell Biol.

[2] Lu LY, Wood JL, Ye L, Minter-Dykhouse K, Saunders TL, Yu X, Chen J (2008). Aurora A is essential for early embryonic development and tumor suppression. J Biol Chem.

[3] Cowley DO, Rivera-Pérez JA, Schliekelman M, He YJ, Oliver TG, Lu L, O'Quinn R, Salmon ED, Magnuson T, Van Dyke T (2009). Aurora-A kinase is essential for bipolar spindle formation and early development. Mol Cell Biol.

[4] Torchia EC, Zhang L, Huebner AJ, Sen S, Roop DR (2013). Aurora kinase-A deficiency during skin development impairs cell division and stratification. J Invest Dermatol.

[5] Yang KT, Li SK, Chang CC, Tang CJ, Lin YN, Lee SC, Tang TK (2010). Aurora-C kinase deficiency causes cytokinesis failure in meiosis I and production of large polyploid oocytes in mice. Mol Biol Cell.

[6] Yoon Y, Cowley DO, Gallant J, Jones SN, Van Dyke T, Rivera-Pérez JA (2012). Conditional Aurora A deficiency differentially affects early mouse embryo patterning. Dev Biol.

[7] Gurden MD, Anderhub SJ, Faisal A, Linardopoulos S (2016). Aurora B prevents premature removal of spindle assembly checkpoint proteins from the kinetochore: A key role for Aurora B in mitosis. Oncotarget.

[8] Cirak Y, Furuncuoglu Y, Yapicier O, Aksu A, Cubukcu E (2015). Aurora A overexpression in breast cancer patients induces taxane resistance and results in worse prognosis. J BUON.

[9] Ferchichi I, Sassi Hannachi S, Baccar A, Marrakchi Triki R, Cremet JY, Ben Romdhane K, Prigent C, Ben Ammar El Gaaied A (2013). Assessment of Aurora A kinase expression in breast cancer: a tool for early diagnosis?. Dis Markers.

[10] Zhang Y, Jiang C, Li H, Lv F, Li X, Qian X, Fu L, Xu B, Guo X (2015). Elevated Aurora B expression contributes to chemoresistance and poor prognosis in breast cancer. Int J Clin Exp Pathol.

[11] Zekri A, Lesan V, Ghaffari SH, Tabrizi MH, Modarressi MH (2012). Gene amplification and overexpression of Aurora-C in breast and prostate cancer cell lines. Oncol Res.

[12] Do TV, Xiao F, Bickel LE, Klein-Szanto AJ, Pathak HB, Hua X, Howe C, O'Brien SW, Maglaty M, Ecsedy JA, Litwin S, Golemis EA, Schilder RJ (2014). Aurora kinase A mediates epithelial ovarian cancer cell migration and adhesion. Oncogene.

[13] Yang G, Chang B, Yang F, Guo X, Cai KQ, Xiao XS, Wang H, Sen S, Hung MC, Mills GB, Chang S, Multani AS, Mercado-Uribe I, Liu J (2010). Aurora kinase A promotes ovarian tumorigenesis through dysregulation of the cell cycle and suppression of BRCA2. Clin Cancer Res.

[14] Davidson B, Nymoen DA, Elgaaen BV, Staff AC, Tropé CG, Kærn J, Reich R, Falkenthal TE (2014). BUB1 mRNA is significantly co-expressed with AURKA and AURKB mRNA in advanced-stage ovarian serous carcinoma. Virchows Arch.

[15] Honma K, Nakanishi R, Nakanoko T, Ando K, Saeki H, Oki E, Iimori M, Kitao H, Kakeji Y, Maehara Y (2014). Contribution of Aurora-A and -B expression to DNA aneuploidy in gastric cancers. Surg Today.

[16] Katsha A, Belkhiri A, Goff L, El-Rifai W (2015). Aurora kinase A in gastrointestinal cancers: time to target. Mol Cancer.

[17] Casorzo L, Dell'Aglio C, Sarotto I, Risio M (2015). Aurora kinase A gene copy number is associated with the malignant transformation of colorectal adenomas but not with the serrated neoplasia progression. Hum Pathol.

[18] Tuncel H, Shimamoto F, Kaneko Guangying Qi H, Aoki E, Jikihara H, Nakai S, Takata T, Tatsuka M (2012). Nuclear Aurora B and cytoplasmic Survivin expression is involved in lymph node metastasis of colorectal cancer. Oncol Lett.

[19] Hosseini S, Hashemzadeh S, Estiar MA, Ebrahimzadeh R, Fakhree MB, Yousefi B, Sheikholeslami S, Modarresi MH, Sakhinia E (2015). Expression Analysis of Aurora-C and Survivin, Two Testis-Specific Genes, in Patients with Colorectal Cancer. Clin Lab.

[20] Wang X, Lu N, Niu B, Chen X, Xie J, Cheng N (2012). Overexpression of Aurora-A enhances invasion and matrix metalloproteinase-2 expression in esophageal squamous cell carcinoma cells. Mol Cancer Res.

[21] Yang SB, Zhou XB, Zhu HX, Quan LP, Bai JF, He J, Gao YN, Cheng SJ, Xu NZ (2007). Amplification and overexpression of Aurora-A in esophageal squamous cell carcinoma. Oncol Rep.

[22] Lo Iacono M, Monica V, Saviozzi S, Ceppi P, Bracco E, Papotti M, Scagliotti GV (2011). Aurora Kinase A expression is associated with lung cancer histological-subtypes and with tumor de-differentiation. J Transl Med.

[23] Takeshita M, Koga T, Takayama K, Ijichi K, Yano T, Maehara Y, Nakanishi Y, Sueishi K (2013). Aurora-B overexpression is correlated with aneuploidy and poor prognosis in non-small cell lung cancer. Lung Cancer.

[24] Twu NF, Yuan CC, Yen MS, Lai CR, Chao KC, Wang PH, Wu HH, Chen YJ (2009). Expression of Aurora kinase A and B in normal and malignant cervical tissue: high Aurora A kinase expression in squamous cervical cancer. Eur J Obstet Gynecol Reprod Biol.

[25] Fujii S, Srivastava V, Hegde A, Kondo Y, Shen L, Hoshino K, Gonzalez Y, Wang J, Sasai K, Ma X, Katayama H, Estecio MR, Hamilton SR (2015). Regulation of AURKC expression by CpG island methylation in human cancer cells. Tumour Biol.

[26] Toughiri R, Li X, Du Q, Bieberich CJ (2013). Phosphorylation of NuMA by Aurora-A kinase in PC-3 prostate cancer cells affects proliferation, survival, and interphase NuMA localization. J Cell Biochem.

[27] Fadri-Moskwik M, Weiderhold KN, Deeraksa A, Chuang C, Pan J, Lin SH, Yu-Lee LY (2012). Aurora B is regulated by acetylation/deacetylation during mitosis in prostate cancer cells. FASEB J.

[28] Premkumar DR, Jane EP, Pollack IF (2015). Cucurbitacin-I inhibits Aurora kinase A, Aurora kinase B and survivin, induces defects in cell cycle progression and promotes ABT-737-induced cell death in a caspase-independent manner in malignant human glioma cells. Cancer Biol Ther.

[29] Diaz RJ, Golbourn B, Shekarforoush M, Smith CA, Rutka JT (2012). Aurora kinase B/C inhibition impairs malignant glioma growth in vivo. J Neurooncol.

[30] Kim SJ, Jang JE, Cheong JW, Eom JI, Jeung HK, Kim Y, Hwang DY, Min YH (2012). Aurora A kinase expression is increased in leukemia stem cells, and a selective Aurora A kinase inhibitor enhances Ara-C-induced apoptosis in acute myeloid leukemia stem cells. Korean J Hematol.

[31] Hartsink-Segers SA, Zwaan CM, Exalto C, Luijendijk MW, Calvert VS, Petricoin EF, Evans WE, Reinhardt D, de Haas V, Hedtjärn M, Hansen BR, Koch T, Caron HN (2013). Aurora kinases in childhood acute leukemia: the promise of aurora B as therapeutic target. Leukemia.

[32] Tatsuka M, Sato S, Kitajima S, Suto S, Kawai H, Miyauchi M, Ogawa I, Maeda M, Ota T, Takata T (2005). Overexpression of Aurora-A potentiates HRAS-mediated oncogenic transformation and is implicated in oral carcinogenesis. Oncogene.

[33] Pannone G, Hindi SA, Santoro A, Sanguedolce F, Rubini C, Cincione RI, De Maria S, Tortorella S, Rocchetti R, Cagiano S, Pedicillo C, Serpico R, Lo Muzio L, Bufo P (2011). Aurora B expression as a prognostic indicator and possible therapeutic target in oral squamous cell carcinoma. Int J Immunopathol Pharmacol.

[34] Ramani P, Sowa-Avugrah E, May MT (2015). High proliferation index, as determined by immunohistochemical expression of Aurora kinase B and geminin, indicates poor prognosis in neuroblastomas. Virchows Arch.

[35] Goos JA, Coupe VM, Diosdado B, Delis-Van Diemen PM, Karga C, Beliën JA, Carvalho B, van den Tol MP, Verheul HM, Geldof AA, Meijer GA, Hoekstra OS, Fijneman RJ, DeCoDe PET group (2013). Aurora kinase A (AURKA) expression in colorectal cancer liver metastasis is associated with poor prognosis. Br J Cancer.

[36] Knowlton AL, Lan W, Stukenberg PT (2006). Aurora B is enriched at merotelic attachment sites, where it regulates MCAK. Curr Biol.

[37] Goto H, Yasui Y, Nigg EA, Inagaki M (2002). Aurora-B phosphorylates Histone H3 at serine28 with regard to the mitotic chromosome condensation. Genes Cells.

[38] Ouchi M, Fujiuchi N, Sasai K, Katayama H, Minamishima YA, Ongusaha PP, Deng C, Sen S, Lee SW, Ouchi T (2004). BRCA1 phosphorylation by Aurora-A in the regulation of G2 to M transition. J Biol Chem.

[39] Brittle AL, Nanba Y, Ito T, Ohkura H (2007). Concerted action of Aurora B, Polo and NHK-1 kinases in centromere-specific histone 2A phosphorylation. Exp Cell Res.

[40] Fu J, Bian M, Jiang Q, Zhang C (2007). Roles of Aurora kinases in mitosis and tumorigenesis. Mol Cancer Res.

[41] Sugimoto K, Urano T, Zushi H, Inoue K, Tasaka H, Tachibana M, Dotsu M (2002). Molecular dynamics of Aurora-A kinase in living mitotic cells simultaneously visualized with histone H3 and nuclear membrane protein importinalpha. Cell Struct Funct.

[42] Kovarikova V, Burkus J, Rehak P, Brzakova A, Solc P, Baran V (2016). Aurora kinase A is essential for correct chromosome segregation in mouse zygote. Zygote.

[43] Uehara R, Tsukada Y, Kamasaki T, Poser I, Yoda K, Gerlich DW, Goshima G, Aurora B (2013). and Kif2A control microtubule length for assembly of a functional central spindle during anaphase. J Cell Biol.

[44] Giet R, McLean D, Descamps S, Lee MJ, Raff JW, Prigent C, Glover DM (2002). Drosophila Aurora A kinase is required to localize D-TACC to centrosomes and to regulate astral microtubules. J Cell Biol.

[45] Bellanger JM, Gönczy P (2003). TAC-1 and ZYG-9 form a complex that promotes microtubule assembly in C. elegans embryos. Curr Biol.

[46] Kufer TA, Silljé HH, Körner R, Gruss OJ, Meraldi P, Nigg EA (2002). Human TPX2 is required for targeting Aurora-A kinase to the spindle. J Cell Biol.

[47] Tsai MY, Wiese C, Cao K, Martin O, Donovan P, Ruderman J, Prigent C, Zheng Y (2003). A Ran signalling pathway mediated by the mitotic kinase Aurora A in spindle assembly. Nat Cell Biol.

[48] Scrofani J, Sardon T, Meunier S, Vernos I (2015). Microtubule nucleation in mitosis by a RanGTP-dependent protein complex. Curr Biol.

[49] Taguchi S, Honda K, Sugiura K, Yamaguchi A, Furukawa K, Urano T (2002). Degradation of human Aurora-A protein kinase is mediated by hCdh1. FEBS Lett.

[50] Bolton MA, Lan W, Powers SE, McCleland ML, Kuang J, Stukenberg PT (2002). Aurora B kinase exists in a complex with survivin and INCENP and its kinase activity is stimulated by survivin binding and phosphorylation. Mol Biol Cell.

[51] Honda R, Körner R, Nigg EA (2003). Exploring the functional interactions between Aurora B, INCENP, and survivin in mitosis. Mol Biol Cell.

[52] Zhang T, Fields JZ, Opdenaker L, Otevrel T, Masuda E, Palazzo JP, Isenberg GA, Goldstein SD, Brand M, Boman BM (2010). Survivin-induced Aurora-B kinase activation: A mechanism by which APC mutations contribute to increased mitoses during colon cancer development. Am J Pathol.

[53] Jelluma N, Brenkman AB, van den Broek NJ, Cruijsen CW, van Osch MH, Lens SM, Medema RH, Kops GJ (2008). Mps1 phosphorylates Borealin to control Aurora B activity and chromosome alignment. Cell.

[54] Zeitlin SG, Shelby RD, Sullivan KF (2001). CENP-A is phosphorylated by Aurora B kinase and plays an unexpected role in completion of cytokinesis. J Cell Biol.

[55] Shimada M, Goshima T, Matsuo H, Johmura Y, Haruta M, Murata K, Tanaka H, Ikawa M, Nakanishi K, Nakanishi M (2016). Essential role of autoactivation circuitry on Aurora B-mediated H2AX-pS121 in mitosis. Nat Commun.

[56] Minoshima Y, Kawashima T, Hirose K, Tonozuka Y, Kawajiri A, Bao YC, Deng X, Tatsuka M, Narumiya S, May WS, Nosaka T, Semba K, Inoue T (2003). Phosphorylation by aurora B converts MgcRacGAP to a RhoGAP during cytokinesis. Dev Cell.

[57] Carmena M, Wheelock M, Funabiki H, Earnshaw WC (2012). The chromosomal passenger complex (CPC): from easy rider to the godfather of mitosis. Nat Rev Mol Cell Biol.

[58] Tseng TC, Chen SH, Hsu YP, Tang TK (1998). Protein kinase profile of sperm and eggs: cloning and characterization of two novel testis-specific protein kinases (AIE1, AIE2) related to yeast and fly chromosome segregation regulators. DNA Cell Biol.

[59] Li X, Sakashita G, Matsuzaki H, Sugimoto K, Kimura K, Hanaoka F, Taniguchi H, Furukawa K, Urano T (2004). Direct association with inner centromere protein (INCENP) activates the novel chromosomal passenger protein, Aurora-C. J Biol Chem.

[60] Sasai K, Katayama H, Hawke DH, Sen S (2016). Aurora-C Interactions with Survivin and INCENP Reveal Shared and Distinct Features Compared with Aurora-B Chromosome Passenger Protein Complex. PLoS One.

[61] Yan X, Cao L, Li Q, Wu Y, Zhang H, Saiyin H, Liu X, Zhang X, Shi Q, Yu L (2005). Aurora C is directly associated with Survivin and required for cytokinesis. Genes Cells.

[62] Dutertre S, Hamard-Péron E, Cremet JY, Thomas Y, Prigent C (2005). The absence of p53 aggravates polyploidy and centrosome number abnormality induced by Aurora-C overexpression. Cell Cycle.

[63] Sasai K, Katayama H, Stenoien DL, Fujii S, Honda R, Kimura M, Okano Y, Tatsuka M, Suzuki F, Nigg EA, Earnshaw WC, Brinkley WR, Sen S (2004). Aurora-C kinase is a novel chromosomal passenger protein that can complement Aurora-B kinase function in mitotic cells. Cell Motil Cytoskeleton.

[64] Gabillard JC, Ulisse S, Baldini E, Sorrenti S, Cremet JY, Coccaro C, Prigent C, D'Armiento M, Arlot-Bonnemains Y (2011). Aurora-C interacts with and phosphorylates the transforming acidic coiled-coil 1 protein. Biochem Biophys Res Commun.

[65] Bernard M, Sanseau P, Henry C, Couturier A, Prigent C (1998). Cloning of STK13, a third human protein kinase related to Drosophila aurora and budding yeast Ipl1 that maps on chromosome 19q13.3-ter. Genomics.

[66] Bischoff JR, Anderson L, Zhu Y, Mossie K, Ng L, Souza B, Schryver B, Flanagan P, Clairvoyant F, Ginther C, Chan CS, Novotny M, Slamon DJ, Plowman GD (1998). A homologue of Drosophila aurora kinase is oncogenic and amplified in human colorectal cancers. EMBO J.

[67] Kimura M, Matsuda Y, Yoshioka T, Sumi N, Okano Y (1998). Identification and characterization of STK12/Aik2: a human gene related to aurora of Drosophila and yeast IPL1. Cytogenet Cell Genet.

[68] Burum-Auensen E, De Angelis PM, Schjølberg AR, Kravik KL, Aure M, Clausen OP (2007). Subcellular localization of the spindle proteins Aurora A, Mad2, and BUBR1 assessed by immunohistochemistry. J Histochem Cytochem.

[69] Rong R, Jiang LY, Sheikh MS, Huang Y (2007). Mitotic kinase Aurora-A phosphorylates RASSF1A and modulates RASSF1A-mediated microtubule interaction and M-phase cell cycle regulation. Oncogene.

[70] Huang XF, Luo SK, Xu J, Li J, Xu DR, Wang LH, Yan M, Wang XR, Wan XB, Zheng FM, Zeng YX, Liu Q (2008). Aurora kinase inhibitory VX-680 increases Bax/Bcl-2 ratio and induces apoptosis in Aurora-A-high acute myeloid leukemia. Blood.

[71] Yang J, Ikezoe T, Nishioka C, Nobumoto A, Udaka K, Yokoyama A (2013). CD34+/CD38− acute myelogenous leukemia cells aberrantly express Aurora kinase A. Int J Cancer.

[72] Moustafa-Kamal M, Gamache I, Lu Y, Li S, Teodoro JG (2013). BimEL is phosphorylated at mitosis by Aurora A and targeted for degradation by βTrCP1. Cell Death Differ.

[73] Sun J, Knickelbein K, He K, Chen D, Dudgeon C, Shu Y, Yu J, Zhang L (2014). Aurora kinase inhibition induces PUMA via NF-κB to kill colon cancer cells. Mol Cancer Ther.

[74] Zou Z, Yuan Z, Zhang Q, Long Z, Chen J, Tang Z, Zhu Y, Chen S, Xu J, Yan M, Wang J, Liu Q (2012). Aurora kinase A inhibition-induced autophagy triggers drug resistance in breast cancer cells. Autophagy.

[75] Dar AA, Zaika A, Piazuelo MB, Correa P, Koyama T, Belkhiri A, Washington K, Castells A, Pera M, El-Rifai W (2008). Frequent overexpression of Aurora Kinase A in upper gastrointestinal adenocarcinomas correlates with potent antiapoptotic functions. Cancer.

[76] Fenouille N, Tichet M, Dufies M, Pottier A, Mogha A, Soo JK, Rocchi S, Mallavialle A, Galibert MD, Khammari A, Lacour JP, Ballotti R, Deckert M, Tartare-Deckert S (2012). The epithelial-mesenchymal transition (EMT) regulatory factor SLUG (SNAI2) is a downstream target of SPARC and AKT in promoting melanoma cell invasion. PLoS One.

[77] Sengle G, Tsutsui K, Keene DR, Tufa SF, Carlson EJ, Charbonneau NL, Ono RN, Sasaki T, Wirtz MK, Samples JR, Fessler LI, Fessler JH, Sekiguchi K (2012). Microenvironmental regulation by fibrillin-1. PLoS Genet.

[78] Wan XB, Long ZJ, Yan M, Xu J, Xia LP, Liu L, Zhao Y, Huang XF, Wang XR, Zhu XF, Hong MH, Liu Q (2008). Inhibition of Aurora-A suppresses epithelial-mesenchymal transition and invasion by downregulating MAPK in nasopharyngeal carcinoma cells. Carcinogenesis.

[79] Liu X, Li Z, Song Y, Wang R, Han L, Wang Q, Jiang K, Kang C, Zhang Q (2016). AURKA induces EMT by regulating histone modification through Wnt/β-catenin and PI3K/Akt signaling pathway in gastric cancer. Oncotarget.

[80] D'Assoro AB, Liu T, Quatraro C, Amato A, Opyrchal M, Leontovich A, Ikeda Y, Ohmine S, Lingle W, Suman V, Ecsedy J, Iankov I, Di Leonardo A (2014). The mitotic kinase Aurora—a promotes distant metastases by inducing epithelial-to-mesenchymal transition in ERα(+) breast cancer cells. Oncogene.

[81] Zheng F, Yue C, Li G, He B, Cheng W, Wang X, Yan M, Long Z, Qiu W, Yuan Z, Xu J, Liu B, Shi Q (2016). Nuclear AURKA acquires kinase-independent transactivating function to enhance breast cancer stem cell phenotype. Nat Commun.

[82] Zheng FM, Long ZJ, Hou ZJ, Luo Y, Xu LZ, Xia JL, Lai XJ, Liu JW, Wang X, Kamran M, Yan M, Shao SJ, Lam EW (2014). A novel small molecule aurora kinase inhibitor attenuates breast tumor-initiating cells and overcomes drug resistance. Mol Cancer Ther.

[83] Noh EM, Lee YR, Hong OY, Jung SH, Youn HJ, Kim JS (2015). Aurora kinases are essential for PKC-induced invasion and matrix metalloproteinase-9 expression in MCF-7 breast cancer cells. Oncol Rep.

[84] Chefetz I, Holmberg JC, Alvero AB, Visintin I, Mor G (2011). Inhibition of Aurora-A kinase induces cell cycle arrest in epithelial ovarian cancer stem cells by affecting NFĸB pathway. Cell Cycle.

[85] Xia Z, Wei P, Zhang H, Ding Z, Yang L, Huang Z, Zhang N (2013). AURKA governs self-renewal capacity in glioma-initiating cells via stabilization/activation of β-catenin/Wnt signaling. Mol Cancer Res.

[86] González-Loyola A, Fernández-Miranda G, Trakala M, Partida D, Samejima K, Ogawa H, Cañamero M, de Martino A, Martínez-Ramírez Á, de Cárcer G, Pérez de I, Castro I, Earnshaw WC, Malumbres M (2015). Aurora B Overexpression Causes Aneuploidy and p21Cip1 Repression during Tumor Development. Mol Cell Biol.

[87] Guise AJ, Greco TM, Zhang IY, Yu F, Cristea IM (2012). Aurora B-dependent regulation of class IIa histone deacetylases by mitotic nuclear localization signal phosphorylation. Mol Cell Proteomics.

[88] Wang C, Chen J, Cao W, Sun L, Sun H, Liu Y (2016). Aurora-B and HDAC synergistically regulate survival and proliferation of lymphoma cell via AKT, mTOR and Notch pathways. Eur J Pharmacol.

[89] Porcelli L, Guida G, Quatrale AE, Cocco T, Sidella L, Maida I, Iacobazzi RM, Ferretta A, Stolfa DA, Strippoli S, Guida S, Tommasi S, Guida M, Azzariti A (2015). Aurora kinase B inhibition reduces the proliferation of metastatic melanoma cells and enhances the response to chemotherapy. J Transl Med.

[90] Pacchierotti F, Adler ID, Eichenlaub-Ritter U, Mailhes JB (2007). Gender effects on the incidence of aneuploidy in mammalian germ cells. Environ Res.

[91] Liu Q, Kaneko S, Yang L, Feldman RI, Nicosia SV, Chen J, Cheng JQ (2004). Aurora-A abrogation of p53 DNA binding and transactivation activity by phosphorylation of serine 215. J Biol Chem.

[92] Katayama H, Sasai K, Kawai H, Yuan ZM, Bondaruk J, Suzuki F, Fujii S, Arlinghaus RB, Czerniak BA, Sen S (2004). Phosphorylation by aurora kinase A induces Mdm2-mediated destabilization and inhibition of p53. Nat Genet.

[93] Hsueh KW, Fu SL, Huang CY, Lin CH (2011). Aurora-A phosphorylates hnRNPK and disrupts its interaction with p53. FEBS Lett.

[94] Gully CP, Velazquez-Torres G, Shin JH, Fuentes-Mattei E, Wang E, Carlock C, Chen J, Rothenberg D, Adams HP, Choi HH, Guma S, Phan L, Chou PC (2012). Aurora B kinase phosphorylates and instigates degradation of p53. Proc Natl Acad Sci USA.

[95] Yan M, Wang C, He B, Yang M, Tong M, Long Z, Liu B, Peng F, Xu L, Zhang Y, Liang D, Lei H, Subrata S (2016). Aurora-A Kinase: A Potent Oncogene and Target for Cancer Therapy. Med Res Rev.

[96] Li Z, Sun Y, Chen X, Squires J, Nowroozizadeh B, Liang C, Huang J (2015). p53 Mutation Directs AURKA Overexpression via miR-25 and FBXW7 in Prostatic Small Cell Neuroendocrine Carcinoma. Mol Cancer Res.

[97] Teng CL, Hsieh YC, Phan L, Shin J, Gully C, Velazquez-Torres G, Skerl S, Yeung SC, Hsu SL, Lee MH (2012). FBXW7 is involved in Aurora B degradation. Cell Cycle.

[98] Stolz A, Ertych N, Kienitz A, Vogel C, Schneider V, Fritz B, Jacob R, Dittmar G, Weichert W, Petersen I, Bastians H (2010). The CHK2-BRCA1 tumour suppressor pathway ensures chromosomal stability in human somatic cells. Nat Cell Biol.

[99] Ertych N, Stolz A, Valerius O, Braus GH, Bastians H (2016). CHK2-BRCA1 tumor-suppressor axis restrains oncogenic Aurora-A kinase to ensure proper mitotic microtubule assembly. Proc Natl Acad Sci USA.

[100] Sagulenko E, Savelyeva L, Ehemann V, Sagulenko V, Hofmann W, Arnold K, Claas A, Scherneck S, Schwab M (2007). Suppression of polyploidy by the BRCA2 protein. Cancer Lett.

[101] Bodvarsdottir SK, Hilmarsdottir H, Birgisdottir V, Steinarsdottir M, Jonasson JG, Eyfjord JE (2007). Aurora-A amplification associated with BRCA2 mutation in breast tumours. Cancer Lett.

[102] Dutertre S, Cazales M, Quaranta M, Froment C, Trabut V, Dozier C, Mirey G, Bouché JP, Theis-Febvre N, Schmitt E, Monsarrat B, Prigent C, Ducommun B (2004). Phosphorylation of CDC25B by Aurora-A at the centrosome contributes to the G2-M transition. J Cell Sci.

[103] Aradottir M, Reynisdottir ST, Stefansson OA, Jonasson JG, Sverrisdottir A, Tryggvadottir L, Eyfjord JE, Bodvarsdottir SK (2014). Aurora A is a prognostic marker for breast cancer arising in BRCA2 mutation carriers. J Pathol Clin Res.

[104] Yang G, Mercado-Uribe I, Multani AS, Sen S, Shih IM, Wong KK, Gershenson DM, Liu J (2013). RAS promotes tumorigenesis through genomic instability induced by imbalanced expression of Aurora-A and BRCA2 in midbody during cytokinesis. Int J Cancer.

[105] Lu L, Han H, Tian Y, Li W, Zhang J, Feng M, Li Y (2015). Aurora kinase A mediates c-Myc's oncogenic effects in hepatocellular carcinoma. Mol Carcinog.

[106] den_Hollander J, Rimpi S, Doherty JR, Rudelius M, Buck A, Hoellein A, Kremer M, Graf N, Scheerer M, Hall MA, Goga A, von_Bubnoff N, Duyster J (2010). Aurora kinases A and B are up-regulated by Myc and are essential for maintenance of the malignant state. Blood.

[107] Keller UB, Old JB, Dorsey FC, Nilsson JA, Nilsson L, MacLean KH, Chung L, Yang C, Spruck C, Boyd K, Reed SI, Cleveland JL (2007). Myc targets Cks1 to provoke the suppression of p27Kip1, proliferation and lymphomagenesis. EMBO J.

[108] Vlach J, Hennecke S, Alevizopoulos K, Conti D, Amati B (1996). Growth arrest by the cyclin-dependent kinase inhibitor p27Kip1 is abrogated by c-Myc. EMBO J.

[109] Brockmann M, Poon E, Berry T, Carstensen A, Deubzer HE, Rycak L, Jamin Y, Thway K, Robinson SP, Roels F, Witt O, Fischer M, Chesler L, Eilers M (2013). Small molecule inhibitors of aurora-a induce proteasomal degradation of N-myc in childhood neuroblastoma. Cancer Cell.

[110] Niu H, Manfredi M, Ecsedy JA (2015). Scientific Rationale Supporting the Clinical Development Strategy for the Investigational Aurora A Kinase Inhibitor Alisertib in Cancer. Front Oncol.

[111] Yang D, Liu H, Goga A, Kim S, Yuneva M, Bishop JM (2010). Therapeutic potential of a synthetic lethal interaction between the MYC proto-oncogene and inhibition of aurora-B kinase. Proc Natl Acad Sci USA.

[112] Yang J, Ikezoe T, Nishioka C, Udaka K, Yokoyama A (2014). Bcr-Abl activates AURKA and AURKB in chronic myeloid leukemia cells via AKT signaling. Int J Cancer.

[113] Dere R, Perkins AL, Bawa-Khalfe T, Jonasch D, Walker CL (2015). β-catenin links von Hippel-Lindau to aurora kinase A and loss of primary cilia in renal cell carcinoma. J Am Soc Nephrol.

[114] Dutta-Simmons J, Zhang Y, Gorgun G, Gatt M, Mani M, Hideshima T, Takada K, Carlson NE, Carrasco DE, Tai YT, Raje N, Letai AG, Anderson KC, Carrasco DR (2009). Aurora kinase A is a target of Wnt/beta-catenin involved in multiple myeloma disease progression. Blood.

[115] Xu C, Kim NG, Gumbiner BM (2009). Regulation of protein stability by GSK3 mediated phosphorylation. Cell Cycle.

[116] Dar AA, Belkhiri A, El-Rifai W (2009). The aurora kinase A regulates GSK-3beta in gastric cancer cells. Oncogene.

[117] Howe LR, Brown AM (2004). Wnt signaling and breast cancer. Cancer Biol Ther.

[118] Nusse R (2005). Wnt signaling in disease and in development. Cell Res.

[119] Romain C, Paul P, Kim KW, Lee S, Qiao J, Chung DH (2014). Targeting Aurora kinase-A downregulates cell proliferation and angiogenesis in neuroblastoma. J Pediatr Surg.

[120] Zhu XP, Liu ZL, Peng AF, Zhou YF, Long XH, Luo QF, Huang SH, Shu Y (2014). Inhibition of Aurora-B suppresses osteosarcoma cell migration and invasion. Exp Ther Med.

[121] Bavetsias V, Linardopoulos S (2015). Aurora Kinase Inhibitors: Current Status and Outlook. Front Oncol.

[122] Yang J, Ikezoe T, Nishioka C, Tasaka T, Taniguchi A, Kuwayama Y, Komatsu N, Bandobashi K, Togitani K, Koeffler HP, Taguchi H, Yokoyama A (2007). AZD1152, a novel and selective aurora B kinase inhibitor, induces growth arrest, apoptosis, and sensitization for tubulin depolymerizing agent or topoisomerase II inhibitor in human acute leukemia cells in vitro and in vivo. Blood.

[123] Yang Y, Shen Y, Li S, Jin N, Liu H, Yao X (2012). Molecular dynamics and free energy studies on Aurora kinase A and its mutant bound with MLN8054: insight into molecular mechanism of subtype selectivity. Mol Biosyst.

[124] Wang X, Sinn AL, Pollok K, Sandusky G, Zhang S, Chen L, Liang J, Crean CD, Suvannasankha A, Abonour R, Sidor C, Bray MR, Farag SS (2010). Preclinical activity of a novel multiple tyrosine kinase and aurora kinase inhibitor, ENMD-2076, against multiple myeloma. Br J Haematol.

[125] Melichar B, Adenis A, Lockhart AC, Bennouna J, Dees EC, Kayaleh O, Obermannova R, DeMichele A, Zatloukal P, Zhang B, Ullmann CD, Schusterbauer C (2015). Safety and activity of alisertib, an investigational aurora kinase A inhibitor, in patients with breast cancer, small-cell lung cancer, non-small-cell lung cancer, head and neck squamous-cell carcinoma, and gastro-oesophageal adenocarcinoma: a five-arm phase 2 study. Lancet Oncol.

[126] Barr PM, Li H, Spier C, Mahadevan D, LeBlanc M, Ul Haq M, Huber BD, Flowers CR, Wagner-Johnston ND, Horwitz SM, Fisher RI, Cheson BD, Smith SM (2015). Phase II Intergroup Trial of Alisertib in Relapsed and Refractory Peripheral T-Cell Lymphoma and Transformed Mycosis Fungoides: SWOG 1108. J Clin Oncol.

[127] Löwenberg B, Muus P, Ossenkoppele G, Rousselot P, Cahn JY, Ifrah N, Martinelli G, Amadori S, Berman E, Sonneveld P, Jongen-Lavrencic M, Rigaudeau S, Stockman P (2011). Phase 1/2 study to assess the safety, efficacy, and pharmacokinetics of barasertib (AZD1152) in patients with advanced acute myeloid leukemia. Blood.

[128] Kantarjian HM, Martinelli G, Jabbour EJ, Quintás-Cardama A, Ando K, Bay JO, Wei A, Gröpper S, Papayannidis C, Owen K, Pike L, Schmitt N, Stockman PK, Giagounidis A, SPARK-AML1 Investigators (2013). Stage I of a phase 2 study assessing the efficacy, safety, and tolerability of barasertib (AZD1152) versus low-dose cytosine arabinoside in elderly patients with acute myeloid leukemia. Cancer.

[129] Dent SF, Gelmon KA, Chi KN, Jonker DJ, Wainman N, Capier CA, Chen EX, Lyons JF, Seymour L (2013). NCIC CTG IND.181: phase I study of AT9283 given as a weekly 24 hour infusion in advanced malignancies. Invest New Drugs.

[130] Foran J, Ravandi F, Wierda W, Garcia-Manero G, Verstovsek S, Kadia T, Burger J, Yule M, Langford G, Lyons J, Ayrton J, Lock V, Borthakur G (2014). A phase I and pharmacodynamic study of AT9283, a small-molecule inhibitor of aurora kinases in patients with relapsed/refractory leukemia or myelofibrosis. Clin Lymphoma Myeloma Leuk.

[131] Seymour JF, Kim DW, Rubin E, Haregewoin A, Clark J, Watson P, Hughes T, Dufva I, Jimenez JL, Mahon FX, Rousselot P, Cortes J, Martinelli G (2014). A phase 2 study of MK-0457 in patients with BCR-ABL T315I mutant chronic myelogenous leukemia and philadelphia chromosome-positive acute lymphoblastic leukemia. Blood Cancer J.

[132] Soncini C, Carpinelli P, Gianellini L, Fancelli D, Vianello P, Rusconi L, Storici P, Zugnoni P, Pesenti E, Croci V, Ceruti R, Giorgini ML, Cappella P (2006). PHA-680632, a novel Aurora kinase inhibitor with potent antitumoral activity. Clin Cancer Res.

[133] Geuns-Meyer S, Cee VJ, Deak HL, Du B, Hodous BL, Nguyen HN, Olivieri PR, Schenkel LB, Vaida KR, Andrews P, Bak A, Be X, Beltran PJ (2015). Discovery of N-(4-(3-(2-aminopyrimidin-4-yl)pyridin-2-yloxy)phenyl)-4-(4-methylthiophen-2-yl)phthalazin-1-amine (AMG 900), a highly selective, orally bioavailable inhibitor of aurora kinases with activity against multidrug-resistant cancer cell lines. J Med Chem.

[134] Borthakur G, Dombret H, Schafhausen P, Brummendorf TH, Boissel N, Jabbour E, Mariani M, Capolongo L, Carpinelli P, Davite C, Kantarjian H, Cortes JE (2015). A phase I study of danusertib (PHA-739358) in adult patients with accelerated or blastic phase chronic myeloid leukemia and Philadelphia chromosome-positive acute lymphoblastic leukemia resistant or intolerant to imatinib and/or other second generation c-ABL therapy. Haematologica.

[135] Schöffski P, Besse B, Gauler T, de_Jonge MJ, Scambia G, Santoro A, Davite C, Jannuzzo MG, Petroccione A, Delord JP (2015). Efficacy and safety of biweekly i.v. administrations of the Aurora kinase inhibitor danusertib hydrochloride in independent cohorts of patients with advanced or metastatic breast, ovarian, colorectal, pancreatic, small-cell and non-small-cell lung cancer: a multi-tumour, multi-institutional phase II study. Ann Oncol.

[136] Hrabakova R, Kollareddy M, Tyleckova J, Halada P, Hajduch M, Gadher SJ, Kovarova H (2013). Cancer cell resistance to aurora kinase inhibitors: identification of novel targets for cancer therapy. J Proteome Res.

[137] Matulonis UA, Sharma S, Ghamande S, Gordon MS, Del Prete SA, Ray-Coquard I, Kutarska E, Liu H, Fingert H, Zhou X, Danaee H, Schilder RJ (2012). Phase II study of MLN8237 (alisertib), an investigational Aurora A kinase inhibitor, in patients with platinum-resistant or -refractory epithelial ovarian, fallopian tube, or primary peritoneal carcinoma. Gynecol Oncol.

[138] Wilkinson RW, Odedra R, Heaton SP, Wedge SR, Keen NJ, Crafter C, Foster JR, Brady MC, Bigley A, Brown E, Byth KF, Barrass NC, Mundt KE (2007). AZD1152, a selective inhibitor of Aurora B kinase, inhibits human tumor xenograft growth by inducing apoptosis. Clin Cancer Res.

[139] Kollareddy M, Zheleva D, Dzubak P, Brahmkshatriya PS, Lepsik M, Hajduch M (2012). Aurora kinase inhibitors: progress towards the clinic. Invest New Drugs.

[140] Ashton S, Song YH, Nolan J, Cadogan E, Murray J, Odedra R, Foster J, Hall PA, Low S, Taylor P, Ellston R, Polanska UM, Wilson J (2016). Aurora kinase inhibitor nanoparticles target tumors with favorable therapeutic index in vivo. Sci Transl Med.

[141] Xu J, Yue CF, Zhou WH, Qian YM, Zhang Y, Wang SW, Liu AW, Liu Q (2014). Aurora-A contributes to cisplatin resistance and lymphatic metastasis in non-small cell lung cancer and predicts poor prognosis. J Transl Med.

[142] Larsen SL, Yde CW, Laenkholm AV, Rasmussen BB, Duun-Henriksen AK, Bak M, Lykkesfeldt AE, Kirkegaard T (2015). Aurora kinase B is important for antiestrogen resistant cell growth and a potential biomarker for tamoxifen resistant breast cancer. BMC Cancer.

[143] Yoon MJ, Park SS, Kang YJ, Kim IY, Lee JA, Lee JS, Kim EG, Lee CW, Choi KS (2012). Aurora B confers cancer cell resistance to TRAIL-induced apoptosis via phosphorylation of survivin. Carcinogenesis.

[144] Shah NP, Tran C, Lee FY, Chen P, Norris D, Sawyers CL (2004). Overriding imatinib resistance with a novel ABL kinase inhibitor. Science.

[145] Sasaki K, Lahoti A, Jabbour E, Jain P, Pierce S, Borthakur G, Daver N, Kadia T, Pemmaraju N, Ferrajoli A, O'Brien S, Kantarjian H, Cortes J (2016). Clinical Safety and Efficacy of Nilotinib or Dasatinib in Patients With Newly Diagnosed Chronic-Phase Chronic Myelogenous Leukemia and Pre-Existing Liver and/or Renal Dysfunction. Clin Lymphoma Myeloma Leuk.

[146] Yuan H, Wang Z, Zhang H, Roth M, Bhatia R, Chen WY (2012). Overcoming CML acquired resistance by specific inhibition of Aurora A kinase in the KCL-22 cell model. Carcinogenesis.

[147] Illert AL, Seitz AK, Rummelt C, Kreutmair S, Engh RA, Goodstal S, Peschel C, Duyster J, von_Bubnoff N (2014). Inhibition of Aurora kinase B is important for biologic activity of the dual inhibitors of BCR-ABL and Aurora kinases R763/AS703569 and PHA-739358 in BCR-ABL transformed cells. PLoS One.

[148] Opyrchal M, Salisbury JL, Zhang S, McCubrey J, Hawse J, Goetz MP, Lomberk GA, Haddad T, Degnim A, Lange C, Ingle JN, Galanis E, D'Assoro AB (2014). Aurora-A mitotic kinase induces endocrine resistance through down-regulation of ERα expression in initially ERα+ breast cancer cells. PLoS One.

[149] Hole S, Pedersen AM, Lykkesfeldt AE, Yde CW (2015). Aurora kinase A and B as new treatment targets in aromatase inhibitor-resistant breast cancer cells. Breast Cancer Res Treat.

[150] Woo JK, Kang JH, Shin D, Park SH, Kang K, Nho CW, Seong JK, Lee SJ, Oh SH (2015). Daurinol Enhances the Efficacy of Radiotherapy in Lung Cancer via Suppression of Aurora Kinase A/B Expression. Mol Cancer Ther.

[151] Ma Y, Cao H, Lou S, Shao X, Lv W, Qi X, Liu Y, Ying M, He Q, Yang X (2015). Sequential treatment with aurora B inhibitors enhances cisplatin-mediated apoptosis via c-Myc. J Mol Med (Berl).

[152] Tao Y, Zhang P, Frascogna V, Lecluse Y, Auperin A, Bourhis J, Deutsch E (2007). Enhancement of radiation response by inhibition of Aurora-A kinase using siRNA or a selective Aurora kinase inhibitor PHA680632 in p53-deficient cancer cells. Br J Cancer.

[153] Venkataraman S, Alimova I, Tello T, Harris PS, Knipstein JA, Donson AM, Foreman NK, Liu AK, Vibhakar R (2012). Targeting Aurora Kinase A enhances radiation sensitivity of atypical teratoid rhabdoid tumor cells. J Neurooncol.

[154] Niermann KJ, Moretti L, Giacalone NJ, Sun Y, Schleicher SM, Kopsombut P, Mitchell LR, Kim KW, Lu B (2011). Enhanced radiosensitivity of androgen-resistant prostate cancer: AZD1152-mediated Aurora kinase B inhibition. Radiat Res.

[155] Nakamura Y, Suganami A, Fukuda M, Hasan MK, Yokochi T, Takatori A, Satoh S, Hoshino T, Tamura Y, Nakagawara A (2014). Identification of novel candidate compounds targeting TrkB to induce apoptosis in neuroblastoma. Cancer Med.

[156] Polacchini A, Albani C, Baj G, Colliva A, Carpinelli P, Tongiorgi E (2016). Combined cisplatin and aurora inhibitor treatment increase neuroblastoma cell death but surviving cells overproduce BDNF. Biol Open.

[157] Hoellein A, Pickhard A, von_Keitz F, Schoeffmann S, Piontek G, Rudelius M, Baumgart A, Wagenpfeil S, Peschel C, Dechow T, Bier H, Keller U (2011). Aurora kinase inhibition overcomes cetuximab resistance in squamous cell cancer of the head and neck. Oncotarget.

[158] Tanaka S, Arii S, Yasen M, Mogushi K, Su NT, Zhao C, Imoto I, Eishi Y, Inazawa J, Miki Y, Tanaka H (2008). Aurora kinase B is a predictive factor for the aggressive recurrence of hepatocellular carcinoma after curative hepatectomy. Br J Surg.

[159] Mignogna C, Staropoli N, Botta C, De_Marco C, Rizzuto A, Morelli M, Di_Cello A, Franco R, Camastra C, Presta I, Malara N, Salvino A, Tassone P (2016). Aurora Kinase A expression predicts platinum-resistance and adverse outcome in high-grade serous ovarian carcinoma patients. J Ovarian Res.

